# A recent overview on ginsenosides as microRNA modulators in the treatment of human diseases

**DOI:** 10.17179/excli2021-4200

**Published:** 2021-09-27

**Authors:** Tae Kyung Hyun

**Affiliations:** 1 Department of Industrial Plant Science and Technology, College of Agricultural, Life and Environmental Sciences, Chungbuk National University, Cheongju 28644, Republic of Korea

## ⁯

***Dear Editor,***

MicroRNAs (miRNAs) are short (20-22 nucleotides) and highly conserved noncoding transcripts that play a crucial role in the regulation of gene expression, guiding the RNA-induced silencing complex to target mRNAs (Treiber et al., 2019[26]). Under normal physiological conditions, miRNAs are involved in feedback and feedforward loops, which have widespread functions in diverse biological processes, including cell proliferation, differentiation, and apoptosis (Tsang et al., 2007[27]; Reddy, 2015[23]). Since the human disease-related miR15 and miR16, located at chromosome 13q14, were first characterized in chronic lymphocytic leukemia (Calin et al., 2002[4]), much attention has been directed towards the function of miRNAs in a number of disorders such as cancer, viral infections, diabetes, immune-related diseases, and neurodegenerative disorders (Condrat et al., 2020[12]). In addition, accumulating evidence suggests that miRNA-mediated control of gene expression is important for the treatment of various diseases (Ali Syeda et al., 2020[1]; Condrat et al., 2020[12]; Zhang et al., 2020[37]; Wang et al., 2021[31]).

Ginsenosides are a class of steroid glycosides and triterpene saponins that account for the medical effects of ginseng (*Panax ginseng*). Among more than a hundred ginsenosides in ginseng, the most abundant ginsenosides are Rb1, Rb2, Rc, Rd, Re, and Rg1, all of which belong to the protopanaxadiol or protopanaxatriol saponins (Chen et al., 2019[5]). A growing body of evidence indicates that ginsenosides act as antioxidant, antimicrobial, anti-inflammatory, anti-cancer, anti-diabetic, and anti-aging agents, although each ginsenoside exhibits a different pharmacological action (Bai et al., 2018[2]; Zheng et al., 2018[39]; Wang and Roh, 2020[29]). The molecular targets of these effects contain various signaling pathways, including the Ras/Raf/MEK/ERK, PI3K/Akt, NF-κB, and PPARγ/HO-1 signaling pathways (Bai et al., 2018[2]; Zheng et al., 2018[38]). In addition, increasing focus on ginsenosides as miRNA modulators continues to contribute to advances in clinical trials.

In this letter, we present a review of recent clinical findings on the miRNA-mediated pharmacological role of ginsenosides (Table 1(Tab. 1); References in Table 1: Cai et al., 2019[3]; Cheng and Xing, 2019[10]; Chen et al., 2018[6][7], 2019[9], 2021[8]; Chu et al., 2019[11]; Gao and Zheng, 2018[13]; Jia et al., 2019[14]; Jiang et al., 2021[15]; Kim et al., 2017[17], 2021[16]; Lee et al., 2020[18]; Li et al., 2019[19]; Liang et al., 2019[20]; Liu et al., 2020[21]; Paik et al., 2019[22]; Shi et al., 2018[25], 2019[24]; Wang et al., 2018[30], 2021[28]; Wilkes et al., 2021[32]; Xue et al., 2018[33]; Yang et al., 2019[34]; Yi et al., 2019[35]; Yu et al., 2018[36]; Zheng et al., 2018[38]; Zhou et al., 2018[40]). We believe that this letter provides a solid foundation for further evaluation of ginsenosides as miRNA modulators in the prevention and treatment of a number of chronic diseases in humans.

## Conflict of interest

The authors declare no conflict of interest.

## Figures and Tables

**Table 1:**
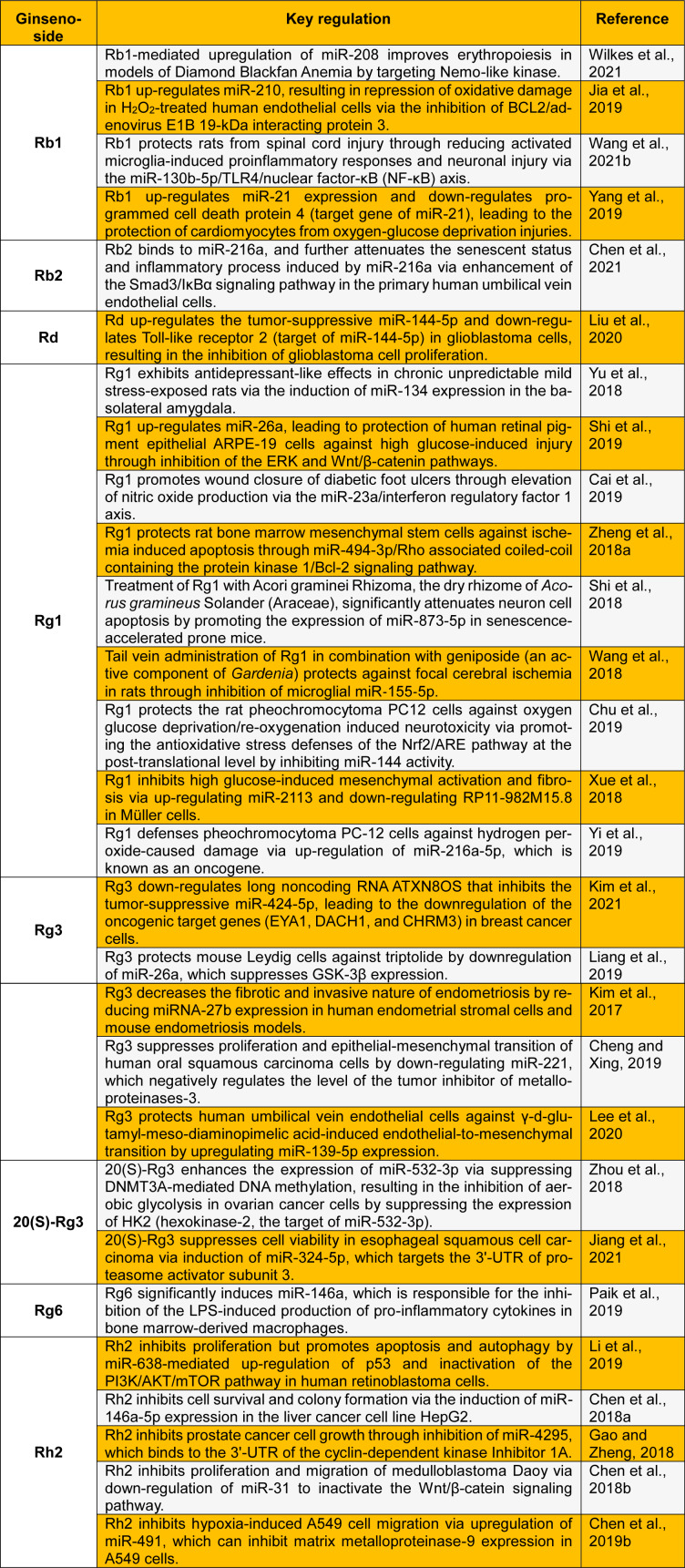
Recent studies on the modulation of microRNAs by ginsenosides as potential therapeutics
